# Influence of *cytochrome P450 (CYP) 2C8* polymorphisms on the efficacy and tolerability of artesunate‐amodiaquine treatment of uncomplicated *Plasmodium falciparum* malaria in Zanzibar

**DOI:** 10.1186/s12936-021-03620-6

**Published:** 2021-02-15

**Authors:** Leyre Pernaute-Lau, Ulrika Morris, Mwinyi Msellem, Andreas Mårtensson, Anders Björkman, Jose Pedro Gil

**Affiliations:** 1 Department of Microbiology, Tumor and Cell biology, Karolinska Institutet, Stockholm, Sweden; 2 BioISI – Biosystems & Integrative Sciences Institute, Faculty of Sciences, University of Lisbon, 1749-016 Lisbon, Portugal; 3 Training and Research, Mnazi Mmoja Hospital, Zanzibar, Tanzania; 4 Department of Women’s and Children’s Health, International Maternal and Child Health (IMCH), Uppsala University, Uppsala, Sweden; 5 Global Health and Tropical Medicine, Instituto de Higiene e Medicina Tropical, Universidade NOVA de Lisboa, 1349-008 Lisbon, Portugal

**Keywords:** *Plasmodium falciparum*, Cytochrome P450, CYP2C8, Artesunate–amodiaquine, Efficacy, Adverse events

## Abstract

**Background:**

The anti-malarial drug, amodiaquine, a commonly used, long-acting partner drug in artemisinin-based combination therapy, is metabolized to active desethyl-amodiaquine (DEAQ) by cytochrome P450 2C8 (CYP2C8). The *CYP2C8* gene carries several polymorphisms including the more frequent minor alleles, CYP2C8*2 and CYP2C8*3. These minor alleles have been associated with decreased enzymatic activity, slowing the amodiaquine biotransformation towards DEAQ. This study aimed to assess the influence of these CYP2C8 polymorphisms on the efficacy and tolerability of artesunate–amodiaquine (AS–AQ) treatment for uncomplicated *Plasmodium falciparum* malaria in Zanzibar.

**Methods:**

Dried blood spots on filter paper were collected from 618 children enrolled in two randomized clinical trials comparing AS–AQ and artemether-lumefantrine in 2002–2005 in Zanzibar. Study participant were under five years of age with uncomplicated falciparum malaria. Human CYP2C8*2 and CYP2C8*3 genotype frequencies were determined by PCR-restriction fragment length polymorphism. Statistical associations between CYP2C8*2 and/or CYP2C8*3 allele carriers and treatment outcome or occurrence of adverse events were assessed by Fisher’s exact test.

**Results:**

The allele frequencies of CYP2C8*2 and CYP2C8*3 were 17.5 % (95 % CI 15.4–19.7) and 2.7 % (95 % CI 1.8–3.7), respectively. There was no significant difference in the proportion of subjects carrying either CYP2C8*2 or CYP2C8*3 alleles amongst those with re-infections (44.1 %; 95 % CI 33.8–54.8) or those with recrudescent infections (48.3 %; 95 % CI 29.4–67.5), compared to those with an adequate clinical and parasitological response (36.7 %; 95 % CI 30.0-43.9) (P = 0.25 and P = 0.31, respectively). However, patients carrying either CYP2C8*2 or CYP2C8*3 alleles were significantly associated with an increased occurrence of non-serious adverse events, when compared with *CYP2C8* *1/*1 wild type homozygotes (44.9 %; 95 % CI 36.1–54.0 vs. 28.1 %; 95 % CI 21.9–35.0, respectively; P = 0.003).

**Conclusions:**

*CYP2C8* genotypes did not influence treatment efficacy directly, but the tolerability to AS–AQ may be reduced in subjects carrying the CYP2C8*2 and CYP2C8*3 alleles. The importance of this non-negligible association with regard to amodiaquine-based malaria chemotherapy warrants further investigation.

## Background

In the mid-1980s, amodiaquine (AQ) was recommended as a malaria prophylaxis for travellers but several reports pointed to high levels of toxicity, mainly agranulocytosis and hepatotoxicity [1, 2], leading to the removal of AQ monotherapy from the Essential Drug List of the World Health Organization (WHO) in 1990 [3]. Some years later, an updated appraisal of available data suggested that AQ toxicity related to severe liver damage and agranulocytosis was primarily seen in non-Africans and, only after several weeks of regular chemoprophylaxis, this drug was reinstated as an option for the treatment of malaria [4, 5]. AQ was reintroduced as an important, slow acting partner drug in artemisinin-based combination therapy (ACT), the current global mainstay for the treatment of uncomplicated falciparum malaria. Nowadays, artesunate–amodiaquine (AS–AQ), a first-generation ACT, is used as first- or second-line treatment in many countries in Africa [6]. AQ is also increasingly used in combination with sulfadoxine-pyrimethamine (SP-AQ) in seasonal malaria chemoprevention, i.e., monthly distribution of intermittent preventative treatment in young children during peak malaria transmission, in several countries of the Sahel sub-region [7, 8]. In numerous clinical trials, AS–AQ efficacy has been high with an estimated mean of 95.1 % cure rate in a large meta-analysis of studies in Africa [9]. Furthermore, treatment (as opposed to prophylaxis) of malaria with AQ has been associated with mild adverse events, including gastrointestinal effects, abdominal pain, neutropenia, nausea, dizziness, and pruritus, but typically not with serious adverse events [4, 10–12].

Amodiaquine is short-lived (half-life 2–8 hours) and is primarily metabolized by cytochrome P450 2C8 (CYP2C8) to its main, biologically active metabolite desethyl-amodiaquine (DEAQ) [13] which has a long terminal elimination half-life (9–18 days) [14]. The main anti-malarial action of AQ is thus carried out by DEAQ, including an initial immediate treatment effect (parasite clearance), as well as a temporary post-treatment protective effect during the elimination phase of the metabolite. The *CYP2C8* gene carries several polymorphisms including the most frequent minor alleles CYP2C8*2 and CYP2C8*3, coding for enzymes with altered activity in comparison with the CYP2C8*1 wild type [15]. The CYP2C8*2 variant has been associated *in vitro* with a sixfold lower AQ metabolism activity than the CYP2C8*1 wild type enzyme [16]. The effect was even greater in the CYP2C8*3 variant, suggesting that any impact of reduced CYP2C8 metabolism would be more pronounced in CYP2C8*3 carriers. CYP2C8*2 is most prevalent in those of African descent, whereas CYP2C8*3 is highly frequent among Caucasians [14, 17–19].

It has been postulated that the impaired conversion of AQ to DEAQ among low activity CYP2C8*2 and CYP2C8*3 carriers is not likely to impact treatment efficacy as both AQ and DEAQ have anti-malarial activity, the latter considered the major active component [16]. However, the prolonged pharmacokinetic profile in poor metabolizers may lead to a non-negligible increased risk of AQ-related adverse events among populations with these specific genotypes [14, 20, 21]. Albeit of interest, only a few studies have investigated the potential association between slow AQ metabolizers and reduced treatment efficacy and/or increased risk of adverse events [16, 21–23]. *In vivo* data on the impact of the low activity CYP2C8*2 allele are sparse, and almost non-existent among CYP2C8*3 carriers due to the very low CYP2C8*3 allele frequency in the generality of African populations, where AS–AQ is primarily used [6, 14].

Zanzibar, where AS–AQ has been first-line treatment for uncomplicated malaria since 2003, has a similar CYP2C8*2 (13.9 %) frequency but higher CYP2C8*3 (2.1 %) allele frequency than most other places in sub-Saharan Africa [16, 18]. This latter particular characteristic sets the opportunity to a more complete investigation of the effect of *CYP2C8* polymorphisms on AQ-based anti-malarial treatment. Therefore, the impact of these *CYP2C8* polymorphisms on treatment outcome and tolerability was retrospectively assessed in two AS–AQ malaria efficacy trials conducted in Zanzibar in 2002–2005, when malaria in these islands was still characterized by high incidence[24, 25]. More specifically, it was assessed if CYP2C8*2 and CYP2C8*3 carriers were at increased risk of new and/or recrudescent infections during the 42-day follow-up period, and if CYP2C8*2 and CYP2C8*3 carriers were at increased risk of experiencing adverse events after AS–AQ treatment.

## Methods

### Study setting and participants

Two randomized clinical trials (ClinicalTrials.gov identifiers: NCT03764527 and NCT03768908) comparing AS–AQ with artemether-lumefantrine (AL) [26–28] were conducted in Zanzibar, Tanzania during 2002–2005 when malaria transmission was high [24, 25] in these islands. Both trials were conducted at Kivunge Hospital, Unguja Island and Micheweni Hospital, Pemba Island and included standard weight-based, three-day supervised treatment courses, with a post-treatment follow-up of 42 days. The AS–AQ PCR-corrected cure rates during the WHO-recommended 28-day follow-up period were 94 and 96 % in the two trials, respectively [28].

**Fig. 1:**
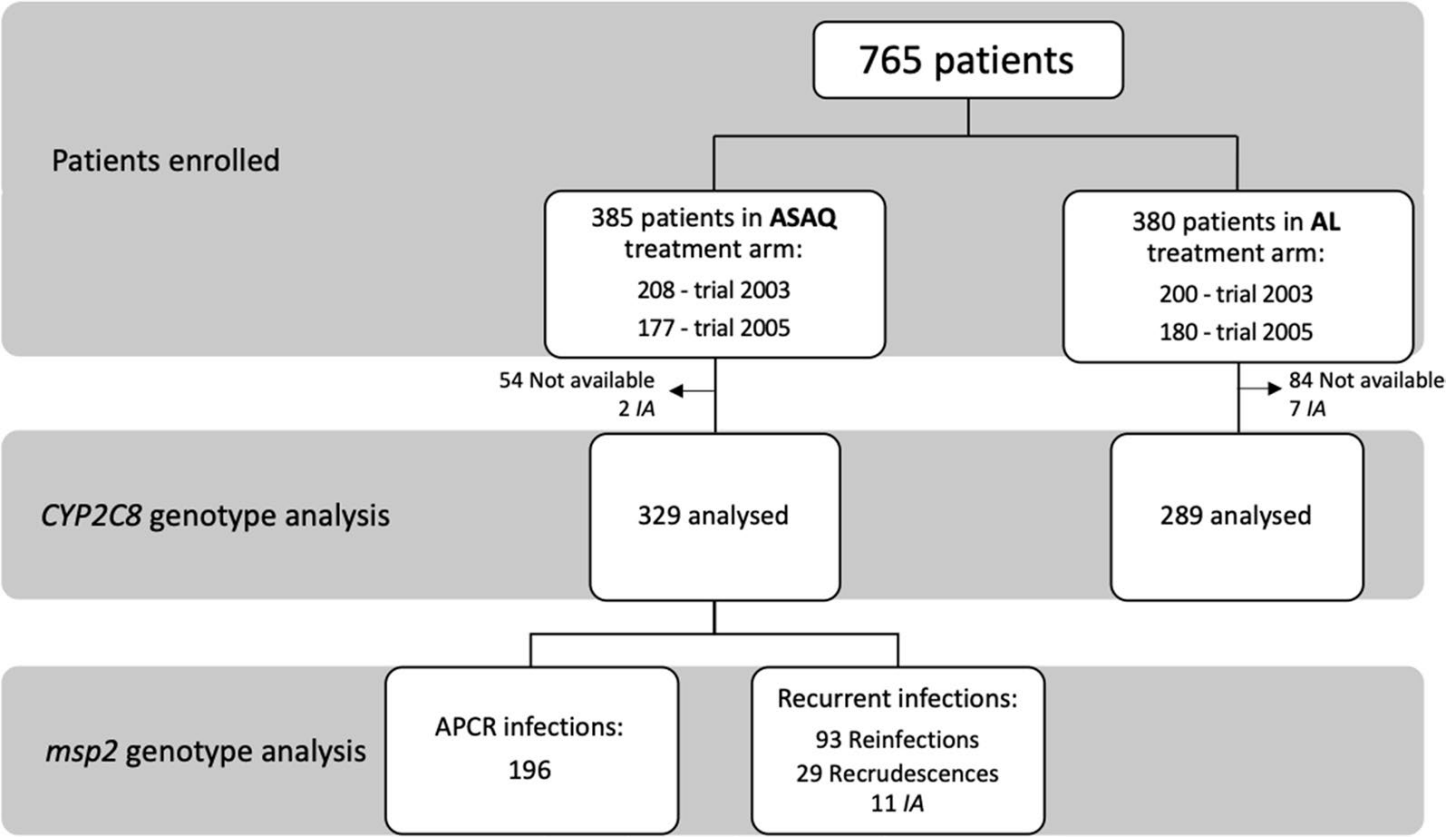
Flow chart of CYPC8*2 and CYPC8*3 genotyping. *AS–AQ* artesunate–amodiaquine, *AL* artemether–lumefantrine, *IA* inconclusive analysis, *ACPR* adequate clinical and parasitological response

CYP2C8*2 and CYP2C8*3 alleles were successfully analysed in 618 malaria-affected children under 5 years of age (Fig. 1). Among these, 329 patients were enrolled in the two AS–AQ clinical trial arms, of which 133 subjects had recurrent infections during post-treatment follow-up, and 196 were selected among the remaining subjects with an adequate clinical and parasitological response (ACPR). In the AL treatment arms of the two clinical trials, 289 subjects were available for *CYP2C8* analysis among the 380 patients enrolled. For the AL-treated subjects, no influence of the *CYP2C8* polymorphisms were expected as CYP2C8 is not involved in the metabolism of either artemether or lumefantrine. These patients were therefore not included in the analyses for treatment outcome but were included as a control in the analysis of adverse events.

### Defining treatment outcome

‘Recurrent infection’ refers to any infection occurring after initial parasite clearance (from day 14) during follow-up. Recurrent infections were defined as either a recrudescent or newly acquired infection by pair-wise molecular analyses of the *Plasmodium falciparum* merozoite surface protein 2 (*pfmsp2*) gene in accordance to WHO guidelines available when the clinical trials were conducted [27]. The size of the *pfmsp2* PCR amplicon for the originally treated infections on day 0 and the day of recurrent parasitaemia were compared by gel electrophoresis [26–28].

### Reporting of adverse events

Non-serious adverse events were defined as any undesirable medical occurrence in a subject during the follow-up and were reported according to perceived severity (mild, moderate, severe) in a case report form for each case. A serious adverse event was defined as an adverse event that resulted in death or was life threatening, an event that required hospitalization, and/or resulted in persistent or significant disability or incapacity. All severe adverse events were associated with clinically suspected severe malaria and thus could not be attributed to the intervention drugs. Therefore, serious adverse events resulting in withdrawal from the clinical trial were excluded from this current study.

### Molecular analysis of CYP2C8*2 and CYP2C8*3

Genomic DNA was extracted by incubation of 3–5 ⌀3 mm punches of peripheral blood samples preserved on Whatman 3MM filter papers at 95°C in 200 µl of PBS. PCR-restriction fragment length polymorphism (PCR-RFLP) was used for the analysis of the CYP2C8*2 I269F (805A > T) and CYP2C8*3 R139K (416A > G) single nucleotide polymorphisms (SNPs). The CYP2C8*3 K399R SNP was not included in the analyses due to the absolute linkage with CYP2C8*3 R139K (R^2^ = 1) [18]. The forward (Fwd) and reverse (Rev) PCR oligonucleotide primers were for **(a)** CYP2C8*2 I269F Fwd 5’-ATGTTGCTCTTACACGAAGTTACA-3’ and Rev 5’-ATCTTACCTGCTCCATTTTGA-3’, and for **(b)** CYP2C8*3 R139K Fwd 5’-CTTCCGTGCTACATGATGACG-3’ and Rev 5’-CTGCTGAGAAAGGCATGAAG-3’. The PCR thermal cycles were: 94°C for 1 min, followed by 40 cycles at 91°C for 30 sec, 62°C for 30 sec, 72°C for 20 sec and 4°C for 10 min. PCR amplifications were followed by discriminative restriction with *BclI* (CYP2C8*2 I269F) and *XmnI* (CYP2C8*3 R139K).

### Defining CYP2C8*2 and CYP2C8*3 genotypes

CYP2C8*1 was defined as the absence of CYP2C8*2 and CYP2C8*3 alleles, i.e., homozygous *1/*1 ‘wild type’ genotypes. CYP2C8*2 carriers included *1/*2 and *2/*2 genotypes and CYP2C8*3 carriers included *1/*3, *3/*3, and *2/*3 genotypes.

### Statistical analysis

Linkage disequilibrium between CYP2C8*3 SNPs was calculated with the LDlink 4.1.0 LDassoc Tool [29]. Allele frequencies and Hardy Weinberg equilibrium were analysed through the Fisher’s exact test. Statistical associations between CYP2C8*2 and/or CYP2C8*3 allele carriers and treatment outcome or adverse events were assessed by Fisher’s exact test. All analyses were performed in STATA/SE version 16.0; statistical significance was defined as P < 0.05.

## Results

### CYP2C8*2 and CYP2C8*3 genotype and allele frequencies in Zanzibar

The CYP2C8*2 and CYP2C8*3 allele frequencies in the studied population were 17.5 % (95 % CI 15.4–19.7) and 2.7 % (95 % CI 1.8–3.7), respectively (Table 1). The proportion of subjects carrying at least one copy of the CYP2C8*2 or the CYP2C8*3 allele were 32.5 % (95 % CI 28.8–36.4) and 4.9 % (95 % CI 3.3–6.6), with 2.9 % (95 % CI 1.7–4.6) of the subjects being homozygous for either the CYP2C8*2 or CYP2C8*3 slow metabolizer alleles. Both alleles were found in Hardy-Weinberg equilibrium with CYP2C8*1 (P = 0.79).

### CYP2C8*2 and CYP2C8*3 genotype frequencies in association to treatment outcome

AS–AQ PCR-corrected cure rates during the WHO-recommended 28-day follow-up period were 94 and 96 % in the two trials, respectively [28]. There was no significant difference in the proportion of subjects carrying the CYP2C8*2 allele among subjects with recurrent infection within the 42-day follow-up in the AS–AQ arms (38.3 %; 95 % CI 30.1–47.2) compared to those with ACPR (31.1 %; 95 % CI 24.7–38.1); P = 0.19 (Table 2). There was also no significant difference in the proportion of subjects carrying the CYP2C8*3 allele in those with recurrent infections (5.3 %; 95 % CI 2.1–10.5) and those with ACPR (5.6 %; 95 % CI 2.8–9.8); P = 1.00.

**Table 1:** CYP2C8 genotype and allele frequencies in Zanzibar

Relative and (absolute) CYP2C8 genotype frequencies		Relative and (absolute) *CYP2C8 *allele frequencies	
2C8*1/2C8*1	0.634 (392)	2C8*1	0.798 (987)
2C8*2/2C8*2	0.024 (15)	2C8*2	0.175 (216)
2C8*3/2C8*3	0.005 (3)	2C8*3	0.027 (33)
2C8*1/2C8*2	0.293 (181)		
2C8*1/2C8*3	0.036 (22)		
2C8*2/2C8*3	0.008 (5)		

Relative and absolute (n) frequencies among 618 children under 5 years old with uncomplicated falciparum malaria. The 2C8*2/2C8*3 genotype are individuals (n=5) that were heterozygous carriers for both CYP2C8*2 and CYP2C8*3. For these, 5 alleles were attributed each to the 2C8*2 and 2C8*3 allele frequencies

Among the 133 recurrent infections in the AS–AQ arm, 122 were successfully PCR-corrected, with 29 recrudescences (clinical failures) and 93 re-infections identified during the 42-day follow-up (Table 2). There was no significant difference in the proportion of subjects carrying either CYP2C8*2 or CYP2C8*3 alleles amongst those with re-infections (44.1 %; 95 % CI 33.8–54.8) or those with recrudescent infections (48.3 %; 95 % CI 29.4–67.5), compared to those with ACPR (36.7 %; 95 % CI 30.0-43.9) (P = 0.25 and P = 0.31, respectively).

### CYP2C8*2 and CYP2C8*3 genotype frequencies in association to occurrence of adverse events

Overall, the AS–AQ treatment was well tolerated. Among all patients, 33 % reported a non-serious adverse event of which 95 % were perceived as mild or moderate and 5 % were perceived as severe. The incidence of adverse events after treatment with AS–AQ was higher in subjects carrying either the CYP2C8*2 or CYP2C8*3 alleles (44.9 %; 95 % CI 36.1–54.0) compared to the incidence in the *CYP2C8* *1/*1 wild type homozygotes (28.1 %; 95 % CI 21.9–35.0) (P = 0.003) (Table 3). No significant difference was observed in the incidence of adverse events after treatment with AL in CYP2C8*2 or CYP2C8*3 carriers (22.1 %; 95 % CI 14.2–31.8) compared to the incidence in the *CYP2C8* *1/*1 wild type homozygotes (23.4 %; 95 % CI 17.6–30.1) (P = 0.88).

**Table 2:** *CYP2C8* genotype frequencies by treatment outcome after treatment with artesunate–amodiaquine

Treatment outcome	*1/*1	*2 carriers	*3 carriers	Total
ACPR; % (n)	63.3 (124)	31.1 (61)	5.6 (11)	100 (196)
Recurrent infections; % (n)	56.4 (75)	38.4 (51)	5.3 (7)	100 (133)
Re-infections; % (n)	55.9 (52)	37.6 (35)	6.5 (6)	100 (93)
Recrudescences; % (n)	51.7 (15)	44.8 (13)	3.5 (1)	100 (29)
Recurrent infections IA; % (n)	72.7 (8)	27.3 (3)	0.0 (0)	100 (11)

Relative (%) and absolute (n) genotype frequencies by treatment outcome among children under 5 years old with uncomplicated falciparum malaria in Zanzibar

*ACPR* adequate clinical and parasitological response, *IA* Inconclusive analysis

## Discussion

CYP2C8*2 and CYP2C8*3 minor allele frequencies were assessed in association to treatment outcome and occurrence of adverse events after anti-malarial treatment in Zanzibar. The observed CYP2C8*3 allele frequency (2.7 %) was consistent with previous reports [18], suggesting that Zanzibar is a region in Africa with relatively high CYP2C8*3 prevalence, compared with other African regions [16, 17, 20]. The CYP2C8*2 allele frequency (17.5 %) is in line with most previous reports from the African continent [16, 19–21, 30, 31], but not as high as was recently reported in Brazzaville, Republic of Congo (37 %) [22]. The present study results show that CYP2C8*2 and CYP2C8*3 carriers were at increased risk of presenting with adverse events after AS–AQ treatment, but without increased risk of experiencing newly acquired or recrudescent *P. falciparum* infections during a 42-day follow-up.

Similarly, no association between CYP2C8*2 heterozygotes and treatment outcome was observed in a study conducted in Burkina Faso, whilst there was an increase in self-reported abdominal pain in CYP2C8*2 heterozygotes but no significant association with other specific adverse events, including nausea, vomiting, fatigue, and jaundice [16]. The observed CYP2C8*3 allele frequency (0.3 %) was too low for any association analyses in that study. Another report, in Ghana, observed a slight but non-significant (P = 0.58) reduction in plasma DEAQ concentrations among subjects with mutant CYP2C8*2 genotypes compared to those with wild-type alleles or heterozygotes [21]. This reduction was however, not associated with treatment outcome or occurrence of adverse events, although the small sample size (N = 81) was lifted as a limiting factor in these analyses. Finally, despite no direct assessments of the association between CYP2C8*2 genotypes and occurrence of adverse events in Congo, the high CYP2C8*2 allele frequency (37 %) reported in Brazzaville has been suggested to have had implications on the choice of first-line treatment in the country [22]. AS–AQ and AL were the first- and second-line treatments, respectively, when ACT was first introduced into the national treatment guidelines in 2006. Interestingly, in 2014 the guidelines were updated with AL as first-line after AS–AQ had been associated with a higher number of drug-related adverse events than AL, possibly due to the high frequency of the CYP2C8*2 allele in the population.

**Table 3:** Incidence of adverse events reported in the artesunate-amodiaquine treatment arm according to *CYP2C8* genotype

	*1/*1	*2 carriers	*3 carriers
Adverse events; % (n)	28.1 (55)	45.9 (50)	38.9 (7)
No adverse events; % (n)	71.9 (141)	54.1 (59)	61.1 (11)
Total; % (n)	100 (196)	100 (109)	100 (18)

Out of the 329 subjects analysed for CYP2C8, 6 had incomplete data regarding adverse events and were therefore excluded from these calculations

Overall, the evidence for the association between CYP2C8*2 and CYP2C8*3 genotypes with AQ and AS–AQ treatment outcome and treatment-associated adverse events is still largely inconclusive, and much hampered by the small sample sizes of previous studies. However, based on the findings of this study, the latter association especially, warrants further investigation. The impact on treatment tolerability may be of particular importance in populations where the CYP2C8*3 allele, having greater impact on reduced CYP2C8 metabolism, is more frequent. The CYP2C8*3 allele has primarily been reported in Caucasian populations [17, 19], which could partly explain the high level of toxicity reported in western travellers after repeated intake of AQ as a malaria prophylaxis. The larger sample size of the current study, together with the relatively high frequency of CYP2C8*3 in Zanzibar, may explain why a significant association between CYP2C8*2 and CYP2C8*3 carriers and occurrence of adverse events was detected in this current study. Indeed, more than 40 % of the CYP2C8*2 and CYP2C8*3 carriers presented with at least one adverse event. This finding might be considered in future pharmacovigilance of treatment with AS–AQ in Zanzibar, seeing that these alleles are present in more than one-third of the population. In addition, future therapeutic efficacy and effectiveness trials of AQ-based treatments in areas with higher prevalence of CYP2C8*2 and CYP2C8*3 genotypes should consider including more detailed pharmacovigilance assessments for further elucidation and confirmation of the association with the occurrence of adverse events, as this could have implications on compliance to the treatment regime.

The relatively high frequency of CYP2C8*3 in Zanzibar may be related to historical links of these islands with Caucasian populations from the Arabian Peninsula. Similar CYP2C8*3 prevalence may be significant in countries along the Sahel region in the northern part of sub-Saharan Africa, bordering the Maghreb countries [32]. Several of these countries (e.g., Chad, Eritrea, Mauritania) use AS–AQ as first-line anti-malarial treatment, and many of them use SP-AQ for seasonal malaria chemoprophylaxis [7]. CYP2C8*3 status may significantly influence treatment tolerability with implications on the uptake of malaria control efforts in these countries.

A relative limitation of this retrospective work is the absence of detailed description of adverse events, including the reporting of causality and concomitant intake of drugs that may act as *CYP2C8* inhibitors and further inhibit enzyme activity [6, 16]. Better pharmacovigilance reporting should be considered for AS–AQ treatment in this specific target population. In addition, individual day-7 DEAQ concentration data could shed better light on this association but such data were not available for the trials conducted in 2002–2005.

## Conclusions

*CYP2C8* genotypes did not appear to influence treatment efficacy directly but the tolerability of AS–AQ may be reduced in subjects carrying the CYP2C8*3 and CYP2C8*2 alleles. The importance of this non-negligible association with regard to AQ-based malaria chemotherapy, particularly in terms of treatment adherence, warrants further investigation.

.

## Data Availability

The datasets used and/or analysed during the current study are available from the corresponding author on reasonable request.

## Abbreviations

WHO: World Health Organization
ACT: Artemisinin-based combination therapy
AS–AQ: Artesunate–amodiaquine
SP-AQ: Sulfadoxine-pyrimethamine plus amodiaquine
CYP2C8: Cytochrome P450 2C8
DEAQ: Desethyl-amodiaquine
AL: Artemether–lumefantrine
ACPR: Adequate clinical and parasitological response
IA: Inconclusive analysis
ZHRC: Zanzibar Health Research Council
*pfmsp2*: *P. falciparum* merozoite surface protein 2
PCR: Polymerase chain reaction
DNA: Deoxyribonucleic acid
RFLP: PCR-restriction fragment length polymorphism
SNP: Single nucleotide polymorphism
CI: Confidence interval
